# Inhibition of TRPC6 by protein kinase C isoforms in cultured human podocytes

**DOI:** 10.1111/jcmm.12660

**Published:** 2015-09-25

**Authors:** Lídia Ambrus, Attila Oláh, Tamás Oláh, György Balla, Moin A. Saleem, Petronella Orosz, Judit Zsuga, Klára Bíró, László Csernoch, Tamás Bíró, Tamás Szabó

**Affiliations:** ^1^ DE‐MTA “Lendület” Cellular Physiology Research GroupDepartment of PhysiologyMedical FacultyUniversity of DebrecenDebrecenHungary; ^2^ Department of PhysiologyMedical FacultyUniversity of DebrecenDebrecenHungary; ^3^ Department of PediatricsMedical FacultyUniversity of DebrecenDebrecenHungary; ^4^ Renal Academic UnitUniversity of BristolBristolUK; ^5^ Department of Health Systems Management and Quality Management for Health CareFaculty of Public HealthUniversity of DebrecenDebrecenHungary; ^6^ Department of ImmunologyMedical FacultyUniversity of DebrecenDebrecenHungary

**Keywords:** human podocytes, transient receptor potential canonical‐6, protein kinase C isoforms, proteinuria

## Abstract

Transient receptor potential canonical‐6 (TRPC6) ion channels, expressed at high levels in podocytes of the filtration barrier, are recently implicated in the pathogenesis of various forms of proteinuric kidney diseases. Indeed, inherited or acquired up‐regulation of TRPC6 activities are suggested to play a role in podocytopathies. Yet, we possess limited information about the regulation of TRPC6 in human podocytes. Therefore, in this study, we aimed at defining how the protein kinase C (PKC) system, one of the key intracellular signalling pathways, regulates TRPC6 function and expression. On human differentiated podocytes, we identified the molecular expressions of both TRPC6 and several PKC isoforms. We also showed that TRPC6 channels are functional since the TRPC6 activator 1‐oleoyl‐2‐acetyl‐sn‐glycerol (OAG) induced Ca^2+^‐influx to the cells. By assessing the regulatory roles of the PKCs, we found that inhibitors of the endogenous activities of classical and novel PKC isoforms markedly augmented TRPC6 activities. In contrast, activation of the PKC system by phorbol 12‐myristate 13‐acetate (PMA) exerted inhibitory actions on TRPC6 and suppressed its expression. Importantly, PMA treatment markedly down‐regulated the expression levels of PKCα, PKCβ, and PKCη reflecting their activation. Taken together, these results indicate that the PKC system exhibits a ‘tonic’ inhibition on TRPC6 activity in human podocytes suggesting that pathological conditions altering the expression and/or activation patterns of podocyte‐expressed PKCs may influence TRPC6 activity and hence podocyte functions. Therefore, it is proposed that targeted manipulation of certain PKC isoforms might be beneficial in certain proteinuric kidney diseases with altered TRPC6 functions.

## Introduction

Transient receptor potential cation channel 6 (TRPC6) is a member of the large TRP channel superfamily. The human TRPC6 is a non‐selective cation channel that is directly activated by diacylglycerol (DAG) in a membrane‐delimited fashion 1, 2, 3. Based on the described activation characteristics, TRPC6 represents a new member of the second‐messenger‐operated cation channels, which are activated by DAG 1. It has been shown that tyrosine phosphorylation of TRPC6 induces a complex formation with phospholipase (PLC‐γ1), which is prerequisite for TRPC6 surface expression. Activation of TRPC6 by DAG analogues, such as 1‐oleoyl‐2‐acetyl‐sn‐glycerol (OAG), results in elevated intracellular Ca^2+^‐level which then triggers multiple downstream signalling events.

Immunofluorescence staining revealed TRPC6 expression throughout the kidney in the glomeruli and tubules 4, 5, 6. Although all cell types within the glomeruli express TRPC6 7, the highest level of expression of TRPC6 was detected in the glomerular epithelial cells, also known as podocytes. In these cells, TRPC6 was shown to be functionally connected to the actin cytoskeleton 8, 9, 10.

Abnormal podocyte function appears to be the final common pathway in a variety of proteinuric kidney diseases 5, 9, 11, 12, 13, 14. Focal segmental glomerulosclerosis (FSGS) is a pathologic entity that is a common cause of nephrotic syndrome in both adults and children. The clinical hallmarks of FSGS include proteinuria, nephrotic syndrome and frequently progressive loss of renal function. Several causative genes encoding mostly regulatory‐ and structural proteins (nephrin, podocin, WT‐1 and α‐actinin‐4) have been identified in the pathogenesis of FSGS with a typical clinical course of therapeutic resistant nephrotic syndrome and associating severe proteinuria 15, 16, 17, 18.

Intriguingly, both genetic and acquired malfunctions of TRPC6 channels were implicated in proteinuric kidney diseases 9, 14, 19, 20. For example, mutation of the *trpc6* gene causes a particularly aggressive form of FSGS 4, 5, 21. The ‘gain‐of‐function’ P112Q mutation in TRPC6 causes enhanced Ca^2+^ entry and a particularly exaggerated response to G‐protein agonists such as angiotensin II 5. Based on *in vitro* and *in vivo* data, it has been suggested that the abnormal TRPC6 function may cause an increase in intracellular Ca^2+^‐level and affects critical interactions with podocyte structural proteins, leading to abnormalities in the slit diaphragm and/or podocyte foot processes 4, 5, 22.

The protein kinase C (PKC) isoenzyme family establishes one of the central regulatory signal transduction pathways involved in practically all major cellular functions. Apparently, the PKC system is also involved in the regulation of kidney functions. For example, PKCα was shown to have a key role in the signalling response after stimulation with transforming growth factor‐β (TGFβ), a protein which promotes podocyte death and development of glomerulosclerosis 23. Others reported the up‐regulation of PKCβ_2_ isoform in human proliferative glomerulonephritis 24. Likewise, up‐regulation of PKCα and β was observed in experimental model of membranous glomerulonephritis 25.

Although (*i*) the PKC activator phorbol 12‐myristate 13‐acetate (PMA) was reported to induce a decrease in TRPC6 expression in kidney mesangial cells which was reversed by the inhibition of nuclear factor kB (NF‐κB) suggesting the involvement of PKCs in this signalling event 26; and (*ii*) in various heterologous systems, PKCs were shown to modulate various TRPs, including TRPC6 27, 28, we lack evidence on the potential of PKC‐dependent regulation of naïve TRPC6 functions in podocytes, especially of human origin. Therefore, in this study, we aimed at investigating the expression and PKC‐dependent functional modulation of TRPC6 channels on human cultured podocytes. In this article, we provide the first evidence that certain PKC isoforms exert a ‘tonic’ inhibitory effect on TRPC6 function suggesting that pathological conditions altering the expression and/or activation patterns of podocyte‐expressed PKCs may influence TRPC6 activity, expression and hence podocyte functions.

## Materials and methods

### Chemicals

The OAG, the PMA, and GF109203X were obtained from Sigma‐Aldrich (St Louis, MO, USA) whereas Gö6983 and Rottlerin were purchased from Calbiochem (Nottingham, UK).

### Antibodies

The following antibodies were employed in this study: rabbit anti‐human TRPC6 (Alomone Labs, Jerusalem, Israel); rabbit anti‐human podocin (Abcam, Cambridge, UK) for immunocytochemistry and another one from Santa Cruz (Heidelberg, Germany) for Western blotting; mouse anti‐human synaptopodin (Progen, Heidelberg, Germany); rabbit anti‐human PKCα, rabbit anti‐human PKCδ and rabbit anti‐human PKCζ (Biomol Res Lab, Plymouth Meeting, PA, USA); rabbit anti‐human PKCλ/ι (Santa Cruz); rabbit anti‐human PKCβ_1_, rabbit anti‐human PKCβ_2_, rabbit anti‐human PKCγ, rabbit anti‐human PKCε, rabbit anti‐human PKCη, rabbit anti‐human PKCθ and rabbit anti‐human β‐actin (Sigma‐Aldrich).

### Cell culturing

**Figure 1:**
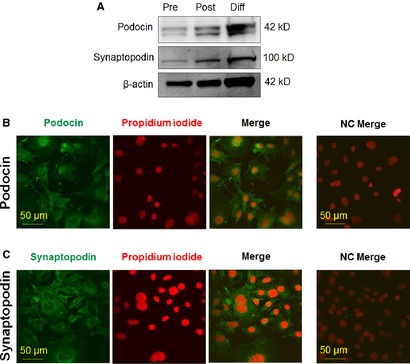
Assessment of *in vitro* differentiation of human podocytes. Expression of differentiation/podocyte markers podocin and synaptopodin as determined by Western blot analysis (**A**) on human podocytes. To assess equal loading, expression of β‐actin was determined. Pre: pre‐confluent (proliferating) culture; Post: post‐confluent (proliferating) culture; Diff: differentiated culture. Podocin (**B**) and synaptopodin (**C**) immunoreactivity was determined on differentiated human podocytes by immunofluorescence labelling (Alexa‐Fluor^®^‐488, green fluorescence). Nuclei were counterstained by propidium iodide (red fluorescence). Calibration mark: 50 μm. NC: negative control.

The human podocyte cell line was established and cultured as described previously 29. In brief, cells were cultured in RPMI‐1640 medium (PAA Laboratories GmbH, Pasching, Austria) supplemented with 10% foetal bovine serum (Invitrogen, Paisley, UK), 50 U/ml penicillin, 50 μg/ml streptomycin, 1.25 μg/ml Fungizone (both from PAA Laboratories GmbH) and insulin‐transferrin‐selenium (1:100; Invitrogen) at 33°C (‘permissive’ conditions). Differentiation was induced by transferring cells to 37°C and kept in culture for 7 days (‘non‐permissive’ conditions). The process of differentiation was assessed using Western blotting and immmunocytochemistry (Figure 1 and Supplementary Figure S1) by determining the expression of the podocyte‐specific marker podocin and the differentiation marker synaptopodin.

### Immunocytochemistry

Human differentiated podocytes were cultured on glass coverslips in 6‐well plates, were fixed by acetone for 5 min. at room temperature, and permeabilized by 0.6% Triton‐X‐100 (Sigma‐Aldrich) in PBS (115 mM NaCl, 20 mM Na_2_PO_4_, pH 7.4; all from Sigma‐Aldrich) for 10 min. Following 30 min. incubation in blocking solution [0.6% Triton‐X‐100 and 1% bovine serum albumin (BSA) containing PBS; Sigma‐Aldrich] at room temperature, cells were probed with the previously mentioned primary antibodies raised against TRPC6 (1:50), podocin (1:100) and synaptopodin (1:100) overnight at 4°C. Following appropriate washing in PBS, coverslips were incubated with Alexa‐488^®^‐conjugated goat anti‐mouse and goat anti‐rabbit secondary antibodies (1:200, Invitrogen) for 1 hr at room temperature. Nuclei were counterstained with propidium‐iodide (Vector Laboratories, Peterborough, UK). Negative control cells were stained omitting the primary antibodies. Visualization of the proteins was performed using Zeiss LSM 510 Meta Confocal Microscope (Zeiss, Oberkochen, Germany). The exposure time and all other settings (gain, gamma and intensity of the excitation) were exactly the same in all cases, including the negative controls.

### Western blot

Cells were harvested and homogenized in protease inhibitor cocktail (1:100; Sigma‐Aldrich) containing detergent mixture (50 mM TRIS HCl, 150 mM NaCl, 1% Triton X‐100, 1% Igepal CA 630, 0.5% sodium deoxicholate; Sigma‐Aldrich). Protein concentrations were determined by BCA reagent (Pierce, Rockford, IL, USA) and set to 1 μg/ml. Equal protein samples (6 μg/well) were subjected to SDS‐PAGE (10% Mini Protean TGX gels; Bio‐Rad, Hercules, CA, USA), and transferred to nitrocellulose membranes, by Trans‐Blot^®^ Turbo^™^ Nitrocellulose Transfer Packs and Trans Blot Turbo System (both from Bio‐Rad). Membranes were probed with the corresponding primary antibodies mentioned above (anti‐TRPC6 1:500; anti‐podocin, 1:100; anti‐synaptopodin 1:100; anti‐PKCα 1:100; anti‐PKCβ_1_ 1:100; anti‐PKCβ_2_ 1:100; anti‐PKCγ 1:100; anti‐PKCδ 1:100; anti‐PKCε 1:100; anti‐PKCζ 1:100 anti‐PKCη 1:100; anti‐PKCθ 1:100; anti‐PKCλ/ι 1:50) in 5% milk containing PBS overnight at 4°C. As secondary antibodies, horseradish peroxidase‐conjugated goat anti‐mouse and goat anti‐rabbit IgGs (1:1000; Bio‐Rad) were employed and the immunoreactive bands were visualized by a SuperSignal West Pico Chemiluminescent Substrate‐Enhanced Chemiluminescence kit (Pierce) using LAS‐3000 Intelligent Dark Box (Fuji, Tokyo, Japan) Gel Logic 1500 Imaging System (Kodak, Tokyo, Japan). To assess equal loading, membranes were re‐probed using a rabbit anti‐β‐actin antibody (1:1000; Sigma‐Aldrich). Densitometric analysis was performed using KODAK Molecular Imaging Systems (Eastman Kodak Company, Rochester, NY, USA).

### RT‐PCR

Total RNA was isolated using TRIzol (Invitrogen) as described by Tóth *et al*. 2011 30, and the isolated total RNA was reverse‐transcribed into cDNA and then amplified on a GeneAmp PCR System 2400 DNA Thermal Cycler (Applied Biosystems, Foster City, CA, USA). Primers were synthesized by Integrated DNA Technologies (Leuven, Belgium) (TRPC6, forward: 5′‐TCAATCTGGTGCCGAGTCCAAAGT‐3′, reverse: 5′‐TTTATGGCCCTGGAACAGCTCAGA‐3′, 86 bp; glyceraldehyde‐3‐phosphate dehydrogenase, forward: 5′‐AATGAGCCCCAGCCTTCTCCAT‐3′, reverse: 5′‐AAGGTCGGAGTCAACGGATTTGG‐3′, 322 bp). PCR products were visualized on 1.5% agarose gel with EZ‐Vision DNA Dye (Amresco, Solon, OH, USA) under UV.

### Fluorescent Ca^2+^‐imaging

Fluorescent Ca^2+^‐imaging was performed by applying two complementary approaches. In multi‐well format imaging, according to our previously optimized protocol 31, 32, human podocytes were seeded in 96‐well/clear‐bottom plates (Greiner Bio‐One, Kremsmuenster, Austria) at a density of 20,000 cells per well in podocyte medium and cultured at ‘non‐permissive’ conditions for 7 days. On day 7, the cells were washed once with 1% BSA (Sigma‐Aldrich) and 2.5 mM Probenecid (Sigma‐Aldrich) containing Hank's solution (136.8 mM NaCl, 5.4 mM KCl, 0.34 mM Na_2_HPO_4_, 0.44 mM KH_2_PO_4_, 0.81 mM MgSO_4_, 1.26 mM CaCl_2_, 5.56 mM glucose, 4.17 mM NaHCO_3_, pH 7.2, all from Sigma‐Aldrich) than loaded with 1 μM Fluo‐4 AM (Life Technologies Corporation, Carlsbad, CA, USA) containing Hank's solution (100 μl/well) at 37°C for 30 min. The cells were washed three times with Hank's solution (100 μl/well; in the case of the ‘low Ca^2+^ Hank’ the CaCl_2_ content was supplemented by equimolar glucose). The plates were then placed into a FlexStation II^384^ Fluorescence Imaging Plate Reader (Molecular Devices, Sunnyvale, CA, USA) and changes in [Ca^2+^]_i_ (reflected by changes in fluorescence; λEX = 494 nm, λEM = 516 nm) induced by various concentrations of the drugs were recorded in each well (during the measurement, cells in a given well were exposed to only one given concentration of the agent). Experiments were performed in triplicates and the averaged data (as well as SEM) were used in the calculations.

For single‐cell imaging 32, changes in [Ca^2+^]_i_ were measured using the calcium sensitive fluorescent dye Fura‐2 (Invitrogen) as reported previously 33. In brief, podocytes were seeded to coverslips and incubated with Fura‐2 AM (10 μM) for 1 hr (37°C) in RPMI supplemented with neostigmin to inhibit acethylcholinesterase. Furthermore, cells were equilibrated in normal Tyrode's solution [NTY; 137 mM NaCl, 5.4 mM KCl, 0.5 mM MgCl_2_, 1.8 mM CaCl_2_, 11.8 mM 4‐(2‐hydroxyethyl)‐1‐piperazineethanesulfonic acid (HEPES), 1 g/l glucose, pH 7.4, all from Sigma‐Aldrich] for 30 min. at room temperature. Coverslips with the Fura‐2 loaded cells were then placed on the stage of an inverted fluorescent microscope (Diaphot; Nikon, Tokyo, Japan). Measurements were performed in NTY. Cells were continuously washed with NTY performed with a background perfusion system. Test solutions were directly applied onto the cells through a perfusion capillary tube (Perfusion Pencil^™^; AutoMate Scientific, Inc., San Francisco, CA, USA) with an internal diameter of 250 μm at a 0.35 ml/min. rate, performed with a local perfusion system (Valve Bank^™^ 8 version 2.0; AutoMate Scientific, Inc., Berkeley, CA, USA). All measurements were performed at room temperature. Excitation wavelength was alternated between 340 and 380 nm by a dual wavelength monochromator (Deltascan; Photon Technology International, New Brunswick, NJ, USA), while the emission was monitored at 510 nm performed with a photomultiplier.

### Statistical analysis

When applicable, data were analysed using a two‐tailed unpaired *t*‐test and *P* < 0.05 values were regarded as significant differences.

Further information about the applied methods can be found in the Supplementary Materials an Methods section.

## Results

### TRPC6 channels are functionally expressed on human podocytes

**Figure 2:**
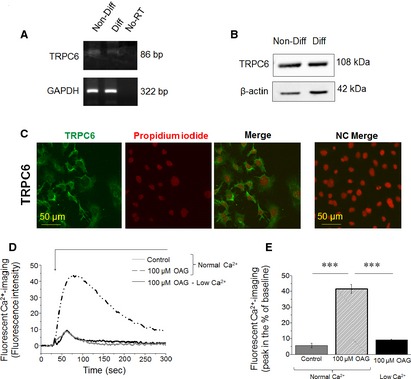
Transient receptor potential canonical‐6 (TRPC6) is expressed and functionally active in human podocytes. (**A**) RT‐PCR analysis of TRPC6 transcripts in non‐differentiated (Non‐Diff) and differentiated (Diff) human podocytes. As an internal control, transcripts of glyceraldehyde‐3‐phosphate dehydrogenase (GAPDH) was determined. No‐RT: ‘no‐RT/water only’ control. (**B**) Western blot analysis of lysates of human non‐differentiated and differentiated podocytes. To assess equal loading, expression of β‐actin was determined. (**C**) Immunocytochemistry. TRPC6‐specific immunoreactivity was determined by immunofluorescence labelling (Alexa‐Fluor^®^‐488, green fluorescence) in human differentiated podocytes. Nuclei were counterstained by Propidium iodide (red fluorescence). Calibration mark: 50 μm. NC: negative control. (**D**) Representative multi‐well fluorescent Ca^2+^‐imaging measurement. Compound was applied as indicated by the bar. OAG: 1‐oleoyl‐acetyl‐sn‐glycerol. (**E**) Statistical analysis of Ca^2+^‐imaging data (*n* = 3 experiments in each case). *** mark significant (*P* < 0.001).

First, we intended to characterize the expression of TRPC6 on cultured, differentiated human podocytes. As revealed by RT‐PCR as well as by Western blotting and immunocytochemistry, TRPC6 channels are expressed by human differentiated podocytes, both at the mRNA and protein levels (Figure 2A–C). Specificity of the TRPC6 antibody was also confirmed by naïve and TRPC6‐overexpressing HEK293 cells (Supplementary Materials and methods; Supplementary Figure S2). We then assessed the functional presence of these channels by employing Fluo 4‐AM‐based Ca^2+^‐imaging. As shown in Figure 2D and E, the TRPC6 activator OAG markedly increased intracellular Ca^2+^‐level of the cells. Importantly, this effect was almost completely abolished by the removal of the extracellular Ca^2+^ suggesting that the OAG induced Ca^2+^‐influx to the cells, most probably *via* TRPC6.

### Human podocytes express multiple PKC isoenzymes

**Figure 3:**
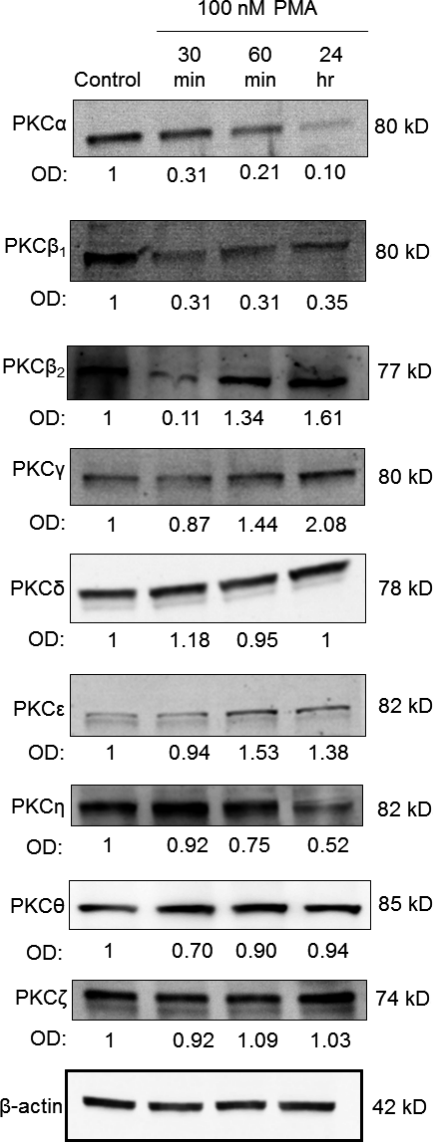
Multiple protein kinase C (PKC) isoforms are expressed and modulated by phorbol 12‐myristate 13‐acetate (PMA) in human podocytes. Human differentiated podocytes were treated with 100 nM PMA for the times indicated (control represents the 0 time‐point) and then Western blot analyses were performed to assess expression levels of the PKC isoenzymes. Immunoreactive bands were then subjected to densitometric analyses and the optical density (OD) values were determined. Numbers indicate OD values compared to those of the control expressions (regarded as 1). For loading control, the expression of β‐actin was determined. These images are representatives from multiple experiments (*n* = 3 experiments in each case except for PKCα, PKCδ and PKCε, where *n* = 6, 4 and 4, respectively).

Since the major goal of this study was to uncover the potential role of the PKC system in the regulation of TRPC6, we then determined the PKC isoform pattern of human differentiated podocytes (which has not been extensively investigated so far). As revealed by Q‐PCR (Supplementary Materials and methods; Supplementary Figure S3) and Western blotting (Figure 3), human podocytes express multiple PKC isoenzymes; namely, the presence of the conventional cPKCα, β_1_, β_2_ and γ; the novel nPKCδ, ε, η and θ; and the atypical aPKCζ were indentified (the expression of aPKCλ/ι was not detected, data not shown).

### Suppression of endogenous PKC activities markedly affects OAG‐induced TRPC6 activation

**Figure 4:**
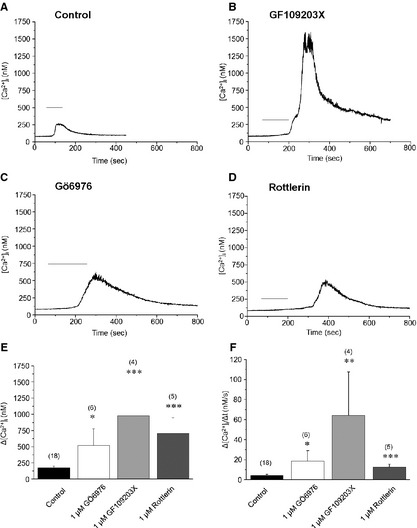
Protein kinase C (PKC) inhibitors augment the transient receptor potential canonical‐6 (TRPC6)‐mediated Ca^2+^‐response in human podocytes. Single‐cell Ca^2+^‐measurements. OAG (200 μM) was applied as indicated by the bars. (**A**) Control conditions (no pretreatment). (**B**–**D**) Effects of pretreatment with the general PKC blocker GF109203X (1 μM, **B**), Gö6976, inhibitor of cPKC isoforms (1 μM, **C**), or Rottlerin, inhibitor of PKCδ (1 μM, **D**) on the OAG‐induced Ca^2+^‐response. (**E** and **F**) Statistical analyses of the above Ca^2+^‐imaging data. Measured peak amplitudes (**E**) and maximal rate of rise of the transients (**F**) were plotted as mean ± SEM of multiple independent determinations as indicated above the corresponding columns. *P < 0.05, **P < 0.01, ***P < 0.001 compared to the control group.

We then assessed whether the modulation of the endogenous activities of these PKC isoforms (especially of those which showed high levels) affect the function of TRPC6. For this, human podocytes were pretreated for 30 min. with GF109203X (inhibitor of the conventional and novel PKCs); Gö6976 (inhibitor of the classical isoforms); and Rottlerin (inhibitor of PKCδ) and then cells were challenged by the TRPC6 activator OAG. As seen in Figure 4, all inhibitors markedly and statistically significantly augmented the effect of OAG to induce TRPC6‐mediated Ca^2+^‐elevation in the cells. These data suggest that, in differentiated human podocytes, the endogenous activities of classical and novel PKCs exert a constitutively present, ‘tonic’ inhibition on TRPC6.

**Figure 5:**
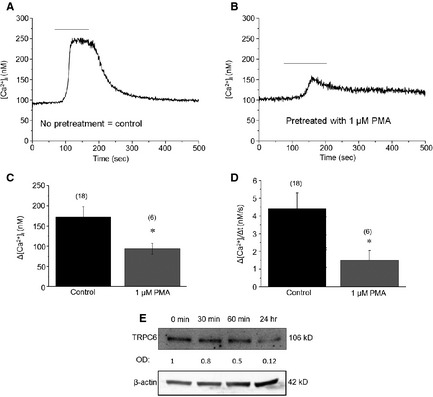
Phorbol 12‐myristate 13‐acetate (PMA) suppresses transient receptor potential canonical‐6 (TRPC6) activity and expression in human podocytes. (**A** and **B**) Single‐cell Ca^2+^‐measurements. OAG (200 μM) was applied as indicated by the bars. (**A**) Control conditions (no pretreatment). (**B**) Effects of 1 μM PMA‐pretreatment (30 min.) on the OAG induced Ca^2+^‐responses. (**C** and **D**) Statistical analysis of the Ca^2+^‐measurement data. Measured peak amplitudes (**C**) and maximal rate of rise of the transients (**D**) were plotted as mean ± SEM of multiple independent determinations as indicated above the corresponding columns. *P < 0.05 compared to the control group. (**E**) Assessment of TRPC6 expression by Western blotting after 100 nM PMA treatment. Immunoreactive bands were subjected to densitometric analyses and the optical density (OD) values were determined. Numbers indicate OD values compared to those of the control expressions (regarded as 1). For loading control, the expression of β‐actin was determined (*n* = 3 experiments).

We then intended to investigate whether the activation of the PKC system (in other words, the exogenous augmentation of activities of the expressed PKC isoforms) also affect TRPC6 function. For this, following the above protocol, podocytes were pre‐incubated with the ‘general’ PKC activator PMA (which activates both conventional and novel PKCs) for 30 min. and then OAG was administered. As expected, in perfect line with data obtained with the PKC inhibitors, PKC activation by PMA treatment significantly abrogated the TRPC6‐mediated Ca^2+^‐signal induced by OAG (Figure 5), which findings further argue for the inhibitory effects of certain PKC isoforms on TRPC6 function.

It is generally accepted that the down‐regulation of PKC isoforms indicates their activation 34, 35. Therefore, to further assess the above proposal, we next investigated whether PMA treatment affects expressions of certain, PMA‐sensitive isoforms. Indeed, PMA markedly down‐regulated the levels of both the conventional PKCα and PKCβ_1_ in a time‐dependent fashion (Figure 3). Interestingly, a marked suppression of expression of PKCβ_2_ was also observed after 30 min.; however, longer treatment with PMA did not decrease the level of this isoform. In addition, PMA‐induced down‐regulation was also observed in the case of the Ca^2+^‐insensitive novel PKCη. Levels of the other PMA‐sensitive isoforms, as well as of the PMA‐insensitive PKCζ, were not suppressed by the phorbol‐ester treatment.

Previous studies on cultured glomerular mesangial cells suggested that PMA treatment suppresses the expression of TRPC6 26, 36. Therefore, we also assessed whether the PMA‐induced ‘functional’ inhibition of TRPC6 is also accompanied by modulating its expression, when performed with Western blotting. Importantly, as shown in Figure 5E, PMA treatment markedly suppressed TRPC6 expression in a similar time‐dependent fashion seen in the cases of PKC isoform down‐regulation.

## Discussion

In current study, we investigated the expression and functional modulation of TRPC6 channels by the PKC system on human cultured podocytes. Here, we provide the first evidence that, among the various PKC isoenzymes expressed by these cells, certain PKC isoforms (PKCα, β_1_, η and possibly β_2_) exert a constitutive ‘tonic’ inhibition on TRPC6 functions expressed by human podocytes. This argument was supported by the following complementary molecular and pharmacological data: (*i*) Inhibition of the endogenous PKC activities by GF109203X (inhibitor of the cPKCs and nPKCs), Gö6986 (inhibitor of the cPKCs) or Rottlerin (inhibitor of PKCδ) all significantly augmented the Ca^2+^‐response induced by OAG, activator of TRPC6 (Figure 4); (*ii*) Conversely, activation of the cPKC and nPKCs by PMA significantly suppressed the TRPC6‐mediated Ca^2+^‐elevation (Figure 5); (*iii*) Treatment of human podocytes by PMA down‐regulated the expression of certain PKCs (*i.e*. of PKCα, β_1_, β_2_, and η), reflecting their activation (Figure 3); and (*iv*) Activation of the PKC system (presumably of the above isoforms) by PMA also resulted in the suppression of TRPC6 expression by podocytes (Figure 5).

As detailed in the Introduction section, both TRPC6 and the PKC systems have been implicated in numerous physiological and pathophysiological regulatory processes of the kidney and its podocytes. However, the relationship between the two key signalling systems was previously described only on cultured kidney mesangial cells where the application of PMA was shown to suppress TRPC6 levels 26. Since, in our present study, the effect of PKC activation on down‐regulating TRPC6 expression was described on human podocytes as well, it is tempting to propose that the PKC system exerts an overall inhibitory effect on kidney TRPC6 signalling.

The major message of our paper is that the above inhibitory action of the PKC system on TRPC6 expressed by human podocytes is not only realized at the molecular expression level but also on the functional level. Moreover, we have also identified certain PKC isoforms that are most likely involved in mediating the ‘tonic’, PKC‐mediated inhibition of TRPC6 function. Interestingly, very similar phenomena were identified on other cell types. On noradrenergic neurons, TRPC6 channel activity, which was markedly augmented by OAG, was significantly inhibited by PKC activation 37. Likewise, Bousquet *et al*. 27 have shown that application of PMA inhibited whereas of GF109203X augmented agonist‐induced, TRPC6‐coupled Ca^2+^‐entry on smooth muscle cells which inhibitory action was suggested to be mediated by the selective involvement of PKCδ. In our hands, on human podocytes, administration of Rottlerin, an inhibitor of PKCδ, significantly augmented the OAG‐induce Ca^2+^‐response (Figure 4); however, PMA treatment did not modify the expression of this isoform (Figure 5). Since Rottlerin was shown to modulate activities of numerous other kinase systems 38, 39, here we cannot exclude the possibility that the effect of Rottlerin to increase TRPC6‐mediated Ca^2+^‐signal is due to its effect on other target(s) than PKCδ.

Our findings might have clinical implications. As detailed above, molecular and/or functional overexpression and/or malfunction of TRPC6 has been observed in a wide variety of genetic and acquired kidney disorders associated with different degrees of proteinuria 4, 5, 9. In these conditions, a pathologically altered, TRPC6‐controlled intracellular Ca^2+^ homeostasis was suggested to play a key role in the development of podocytopathies. Interestingly, in several of these diseases, altered PKC isoform expression patterns were also described. For example, up‐regulation of PKCβ isoform was found in human proliferative glomerulonephritis 24 and of PKCα and β in membranous glomerulonephritis 25. In these studies, however, it was not clearly shown that the elevated PKC levels were also accompanied by increased PKC activities. Likewise, the causative, functional relationship between the pathological expression patterns and levels of TRPC6 and of PKCs is also not obvious.

In this context, our findings that activities of certain PKC isoforms (PKCα, β_1_, β_2_, η) in human cultured podocytes significantly modulate TRPC6 expression and function are of great importance. Along these lines, it is proposed that pathological *in vivo* conditions altering the expression and/or activation patterns of podocyte‐expressed PKCs may influence TRPC6 activity and hence podocyte functions. Therefore, further pre‐clinical and clinical studies are now invited to uncover whether the targeted manipulation of activities of certain PKC isoforms might be beneficial in the therapeutic management of given proteinuric kidney diseases with altered TRPC6 functions.

## Conflicts of interest

The authors confirm no conflicts of interest.

## Author contribution

LA, AO, TO, JZs and PO performed the experiments. LA and TO analysed the data. MAS provided the cells. GyB, LCs, KB, TB and TSz designed the research study. LA, TB and TSz wrote the article which was carefully edited and reviewed by all other authors.

## Supporting information


**Data S1** Supplementary materials and methods and Supplementary Figures S1‐S3.
