# Supplementary material for: Inhibition of TRPC6 by protein kinase C isoforms in cultured human podocytes
Source: J Cell Mol Med. 2015 Sep 25;19(12):2771–9. doi: 10.1111/jcmm.12660 (PMC4687697; doi:10.1111/jcmm.12660)
Supplement: Supplementary file 1 — Data S1 Supplementary materials and methods and Supplementary Figures S1‐S3. [file JCMM-19-2771-s001.pdf]

Supplementary Information (Ambrus et al., Inhibition of TRPC6 by protein kinase C isoforms in cultured human podocytes)

1. Supplementary Materials and Methods

Culturing of Human embryonic kidney (HEK293) cells

Human embryonic kidney (HEK293) cells were cultured in DMEM medium (PAA Laboratories GmbH, Pasching, Austria) supplemented with 10% fetal bovine serum, (Invitrogen, Paisley, UK), 50 U/ml penicillin, 50 μg/ml streptomycin, 1.25 μg/ml Fungizone (both from PPA Laboratories GmbH) and 2mM L-glutamine (Life Technologies Corporation, Carlsbad, CA, USA) under 5% CO_2 at 37°C.

Plasmid DNA Transfection

The transfection was performed using Lipofectamine™ 2000 (Life Technologies Corporation) according to the manufacturer's instructions. HEK293 cells were plated on 35-mm dishes. At cca. 80% confluence, 12 μg plasmid DNA and Lipofectamin transfection reagent was added to the cells for 3.5 hours. After that the medium was changed to the selection medium (basic culture medium supplemented with 750 μg/ml geneticin [Life Technologies Corporation]).

Vector

The TRPC6 overexpressing vector (EX-U0193-M09) was a kind gift from Noémi Kedei (National Institutes for Health, Bethesda, MD, United States).

Quantitative real-time PCR (Q-PCR)

Quantitative real-time PCR was performed on an ABI Prism 7000sequence detection system (Applied Biosystems, Foster City, CA) by using the 50 nuclease assay. Total RNA was isolated with TRIzol (Invitrogen, Paisley, UK) as described by Tóth *et al.* 2011 [30], and the isolated total RNA was reverse-transcribed into cDNA. For the amplification of the cDNA we used the TaqMan primers and probes (PKCα: assay ID Hs00176973_m1; PKCβ: assay ID Hs00176998_m1; PKCγ: assay ID Hs00177010_m1; PKCδ: assay ID Hs00178914_m1; PKCη: assay ID Hs00178933_m1; PKCε: assay ID Hs00178455_m1; PKCζ: assay ID Hs 00177051_m1; PKCθ: assay ID Hs00234704_m1; PKCλ/ι: assay ID Hs00995854_m1) using the TaqMan universal PCR mastermix protocol (Applied Biosystem). As internal control, transcripts of glyceraldehyde 3-phosphate dehydrogenase (assay ID Hs99999905_m1) were determined.

2. Supplementary Figures

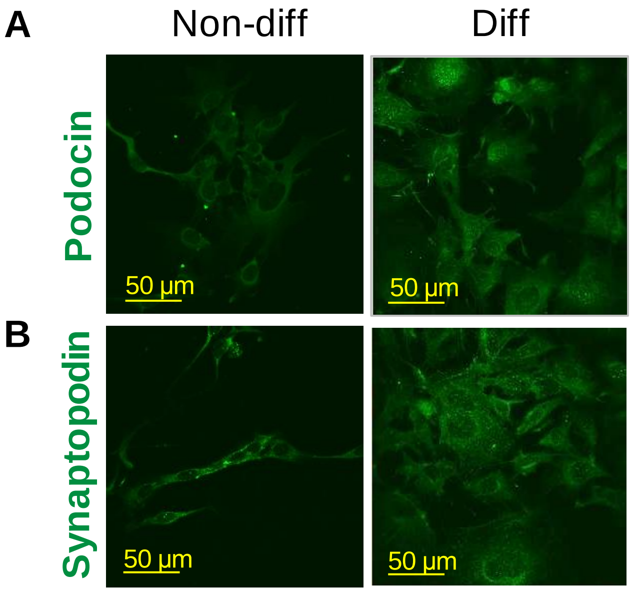

Supplementary Figure S1: *Comparison of podocin and synaptopodin expression of non-differentiated and differentiated podocytes*

Immunoreactivities of podocin (**A**) and synaptopodin (**B**) were determined on non-differentiated (Non-diff) and differentiated (Diff) human podocytes by immunofluorescence labeling (Alexa-Fluor®-488, green fluorescence). Calibration marks: 50 µm. (*Note that images of differentiated podocytes are also shown on* ***Figure 1B*** *and* ***C****!*)

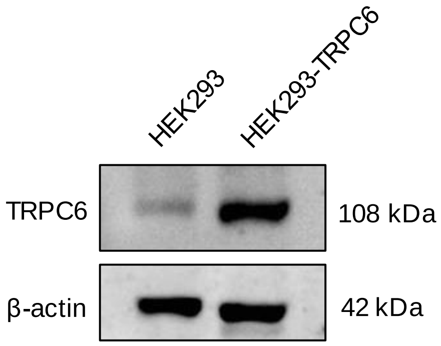

Supplementary Figure S2: *TRPC6 protein expression on TRPC6 overexpressing human embryonic kidney (HEK293) cells*

To assess the specificity of the TRPC6 antibody Western blot analysis of TRPC6 protein was performed in naïve as well as TRPC6-transfected HEK293 cells (HEK293-TRPC6). To assess equal loading, the expression of β-actin was determined.

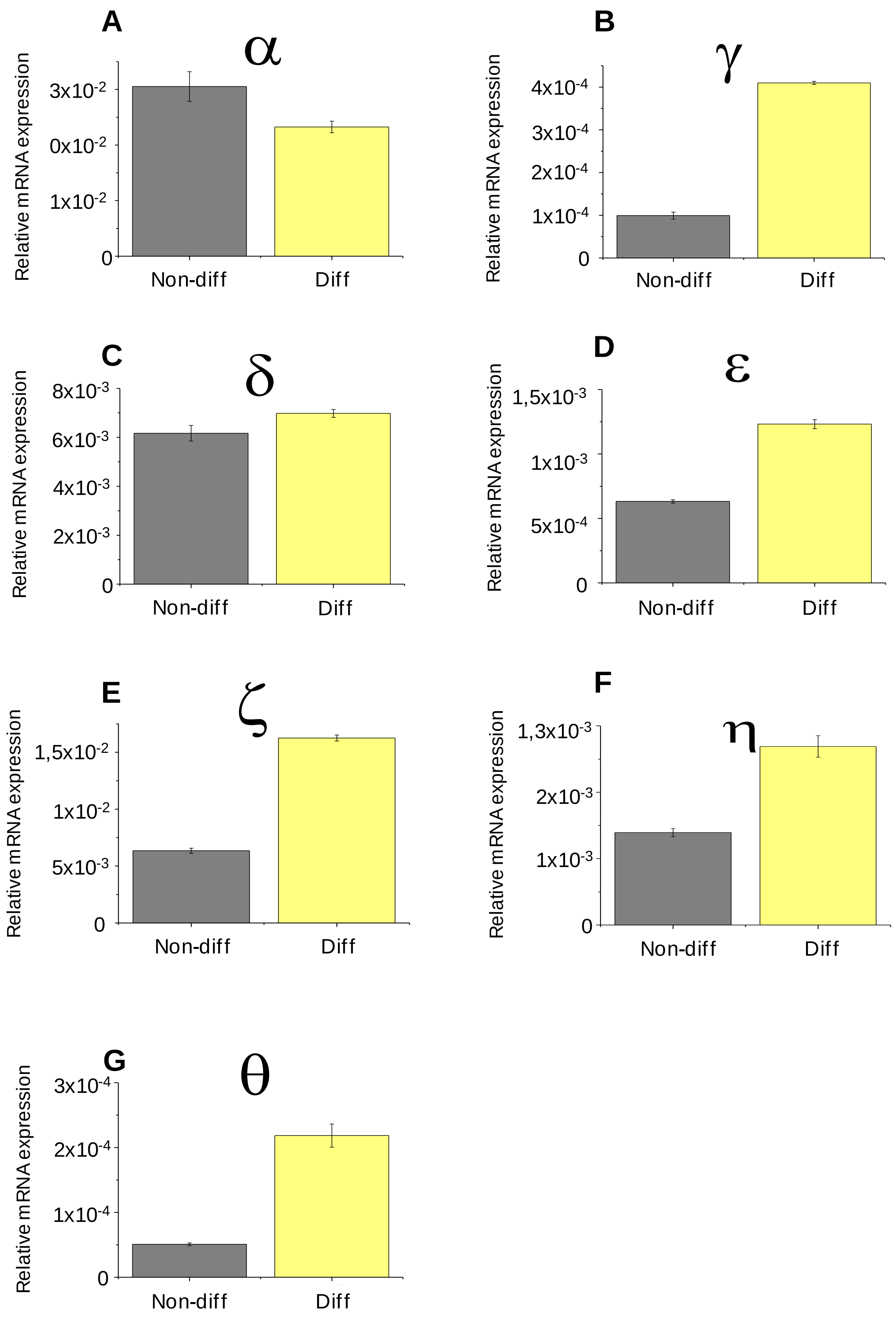

Supplementary Figure S3: *Multiple PKC isoforms are expressed in human podocytes*

The mRNA expression of different PKC isoforms was determined by quantitative real-time PCR on non-differentiated (Non-diff) and differentiated (Diff) human podocytes. PKCα **(A)**, -γ **(B)**, -δ **(C)**, -ε **(D)**, -ζ **(E)**, -η **(F)**, -θ **(G)** were expressed on podocytes. Expressions of PKCβ and –λ/ι was around the detection limit. As an internal control the expression of glyceraldehyde 3-phosphate dehydrogenase was used.
